# Barriers and Enablers for Artificial Intelligence in Dental Diagnostics: A Qualitative Study

**DOI:** 10.3390/jcm10081612

**Published:** 2021-04-10

**Authors:** Anne Müller, Sarah Marie Mertens, Gerd Göstemeyer, Joachim Krois, Falk Schwendicke

**Affiliations:** 1 Department of Oral Diagnostics, Digital Health and Health Services Research, Charité-Universitätsmedizin Berlin, Aßmannshauser Str. 4-6, 14197 Berlin, Germany; anne.mueller@charite.de (A.M.); joachim.krois@charite.de (J.K.); 2 Department of Operative and Preventive Dentistry, Charité-Universitätsmedizin Berlin, Aßmannshauser Str. 4-6, 14197 Berlin, Germany; sarah-marie.mertens@charite.de (S.M.M.); gerd.goestemeyer@charite.de (G.G.)

**Keywords:** artificial intelligence, radiography dental digital, qualitative research, models psychological, models theoretical

## Abstract

The present study aimed to identify barriers and enablers for the implementation of artificial intelligence (AI) in dental, specifically radiographic, diagnostics. Semi-structured phone interviews with dentists and patients were conducted between the end of May and the end of June 2020 (convenience/snowball sampling). A questionnaire developed along the Theoretical Domains Framework (TDF) and the Capabilities, Opportunities and Motivations influencing Behaviors model (COM-B) was used to guide interviews. Mayring’s content analysis was employed to point out barriers and enablers. We identified 36 barriers, conflicting themes or enablers, covering nine of the fourteen domains of the TDF and all three determinants of behavior (COM). Both stakeholders emphasized chances and hopes for AI. A range of enablers for implementing AI in dental diagnostics were identified (e.g., the chance for higher diagnostic accuracy, a reduced workload, more comprehensive reporting and better patient–provider communication). Barriers related to reliance on AI and responsibility for medical decisions, as well as the explainability of AI and the related option to de-bug AI applications, emerged. Decision-makers and industry may want to consider these aspects to foster implementation of AI in dentistry.

## 1. Introduction

The usage of artificial intelligence (AI) for assisting medical diagnostics has been exploding over the last three years [1], and especially in medical image analysis (e.g., radiographs, photos and histological sections) AI applications have proven useful, with accuracies similar to or higher than those for experienced experts [2,3,4]. AI shows further promise [5], including facilitating patient triage [6], supporting decision making [7,8], image post processing [9,10,11], quality control [12] or automated reporting [13]. The uptake of AI technologies in daily care has begun.

In dental radiology, AI has been used for a range of purposes. Detection of dental caries [14,15,16,17], apical lesions [18], periodontal bone loss [19,20,21], tooth fractures [22] or sinusitis [23] is already possible with the use of AI. The reported accuracies for most of these tasks are promising, and the first AI tools for dental diagnostics are currently entering the market.

Notably, medical and dental AI is a complex technology which needs to be properly integrated and handled in the daily workflow. For instance, practitioners need to understand the concept behind most AI tools—supervised machine learning—and they have to be able to interpret AI findings as probabilistic. Aspects such as ethical, safe and explainable AI need to be considered. A range of studies deals with these topics, demonstrating risks of bias and criticizing AI algorithms as black boxes [24,25], which need to be “explained” properly to safeguard them against systematic errors or deviations [26,27,28]. Data protection aspects as well as possible costs and benefits of AI for stakeholders, e.g., providers, patients and payers, need to be reflected and appraised. All these factors jointly will eventually determine how AI applications are accepted, implemented and maintained in clinical practice.

To understand how all these different factors may act as barriers or enablers to implement AI in care, a systematic and comprehensive understanding of stakeholders’ expectations, needs and fears is essential. Qualitative studies can yield such insights but require a theoretical underpinning to ensure the required rigor and to yield the desired understanding [29,30]. Moreover, to foster possible action for improving implementation of AI, researchers should consider multiple dimensions and perspectives of implementation [31,32,33].

In the present qualitative study, we conducted semi-structured qualitative interviews with dentists and patients and used the Theoretical Domains Framework (TDF) [34] to structure and analyze the interviews. The TDF allows the described systematic and comprehensive assessment of various factors associated with behavior, in this case the implementation and use of AI tools for dental diagnostics. Our specific focus in the present study was radiographic diagnostics in dentistry, while notably the identified barriers and enablers likely apply to other dental diagnostics or other use cases in radiology.

## 2. Methods

### 2.1. Research Team and Reflexivity

One author (AM, female) conducted the interviews. AM has a PhD with a background in biology, has been working in healthcare for eight years and has experience in the organization, conduction and analysis of qualitative studies. Together with a second author (FS, male, with longtime experience in qualitative studies and AI applications in dentistry), she conceived the study. There was no relationship established with participants prior to the study. Interview dates were scheduled by a third assisting person. Interviews were carried out from the end of May to the end of June 2020.

### 2.2. Theoretical Framework

The present qualitative study builds on thirteen semi-structured individual interviews with dentists and patients. Interview guides were developed based on TDF [34]. TDF facilitated systematic and comprehensive assessment of attitudes, apprehensions and expectations of dentists and patients concerning dental diagnostics and barriers and enablers for AI-based diagnostic assistance systems. After a thematic content analysis, the Behavior Change Wheel (BCW) and its associated COM-B model (“Capability, Opportunity, Motivation and Behavior”) [35] were applied, allowing to classify identified themes as barriers, facilitators or conflicting themes. Both TDF and BCW have been employed by a number of studies in dental research [36,37,38,39]. Combined, they present a framework that allows to transparently and reproducibly link behavior determinants and interventions to support behavior change, in our case the adoption of AI tools for dental diagnostics [35].

### 2.3. Participant Selection and Setting

Eight dentists (D1–D8) from eight different German dental practices and five patients (P1–P5) were interviewed. Dentists were identified non-systematically via Internet research while patients were randomly selected from the attending patient pool visiting the Department of Oral Diagnostics, Digital Health and Health Services Research, Charité–Universitätsmedizin Berlin, Germany, for a first assessment, triage or treatment. The dentists, two females and six males, were between 37 and 65 years old. The patients, one female and four males, were between 29 and 38 years old. One interviewer (AM) conducted all thirteen telephone interviews, with the dentists and patients being at home or, for dentists, at their practice.

### 2.4. Data Collection

Interview guides covering the fourteen domains of the TDF were developed by two authors (AM and FS): (1) knowledge; (2) skills; (3) social/professional role and identity; (4) beliefs about capabilities; (5) optimism; (6) beliefs about consequences; (7) reinforcement; (8) intentions; (9) goals; (10) memory, attention and decision processes; (11) environmental context and resources; (12) social influences; (13) emotions; and (14) behavioral regulation. Each domain was addressed by a minimum of one question. Interview guides were pilot tested within the research group.

Following written (e-mail) and verbal information about the study, the interviewees signed a declaration of consent. All interviews were conducted in German. Interviewees could freely discuss any topics directly or indirectly related to the question. Each interview took approximately 30 min and was recorded using a digital voice recorder. In addition to recording, notes were made. Transcripts were not returned to participants for comments or corrections.

The interview was structured in two sections. In the first part, dentists and patients explained how they perceived radiographic diagnostics in dentistry. In the second, main part, the interviewer provided a brief overview about the current state-of-the-art of AI in dental diagnostics and described how an AI-based radiographic diagnostics assistance tool could be used in the daily workflow. From there on, interviewees could express their apprehensions, expectations and thoughts in a dialogue fashion, with the interviewer probing for an understanding of facilitators and barriers towards AI applications in dental practice.

### 2.5. Data Analysis, Findings, Reporting

Interviews were transcribed and analyzed using f4 (dr. dresing & pehl, Marburg, Germany). The coding tree was derived according to the TDF domains. To increase inter- and intra-coder reliability, AM performed transcription and analysis independently and FS reviewed the results. An inductive and deductive content analysis was conducted using Mayring’s principles [40]. The identified themes were then classified as barriers, facilitators or conflicting themes and related to the COM-B model. Themes and quotations were translated into English for publication and were double-checked by back translation. Reporting followed the consolidated criteria for reporting qualitative studies (COREQ) checklist [41]. Ethical approval for the study was obtained from the ethics committee of the Charité (EA4/144/20 and EA4/251/20).

## 3. Results

**Figure 1:**
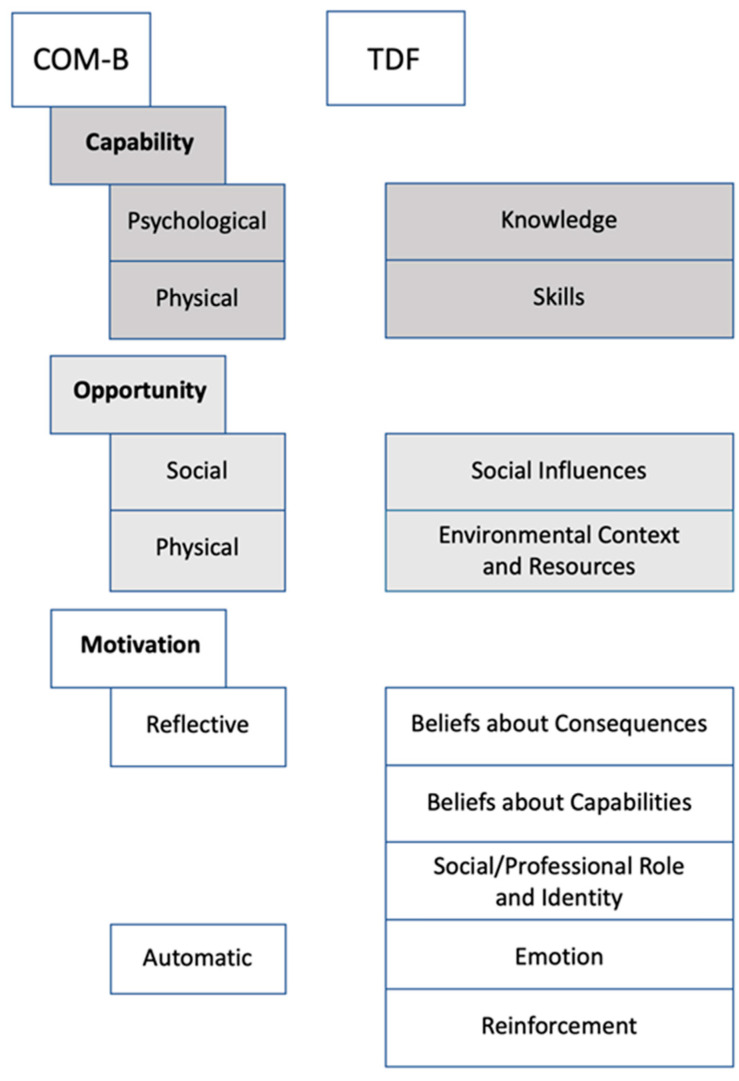
Relation between the TDF and COM-B within the present study.

**Figure 2:**
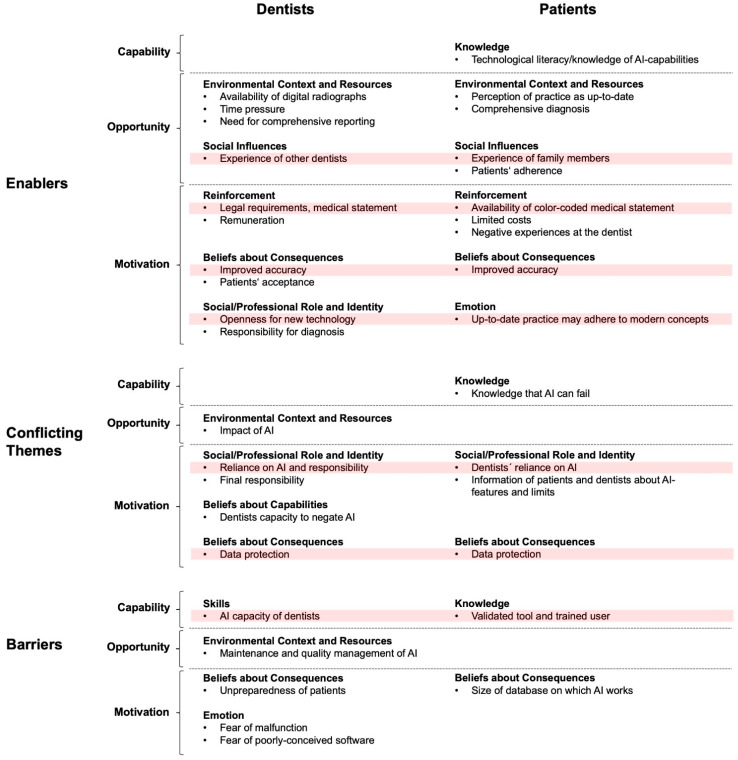
Identified enablers, conflicting themes and barriers for implementation of a new AI-tool in dental X-ray diagnostics. Eight dentists were interviewed, revealing 20 themes for this topic. From five patient interviews, 16 themes were derived. Themes were classified according to the different TDF domains (Knowledge, Environmental Context, Resources, etc.), as well as along the COM-B domains (Capability, Opportunity and Motivation) and as enablers, conflicting themes and barriers. Themes recurring for both dentists and patients are highlighted in pink.

We collected statements from the interviewees and coded them according to the TDF. The identified themes were then organized along the COM-B model. Relevant themes were found in nine domains of the TDF (Figure 1). Figure 2 sums up the identified themes separately for dentists and patients, classified as enablers, conflicting themes and barriers, associated with Capability, Opportunity and Motivation and along the TDF domains.

For dentists, we identified 10 themes as enablers, 5 as conflicting themes and 5 as barriers (in total, 20 themes). For patients, we identified 10 enablers, 4 conflicting themes and 2 barriers (in total, 16 themes; Figure 2). A selection of quotations, illustrating the impact of Capability, Opportunity and Motivation for adopting AI in dental diagnostics is provided in the following sections, first for dentists and then for patients. The sections are organized along enablers, conflicting themes or barriers, and then along the COM as well as TDF domains.

### 3.1. Enablers for Dentists

Availability of digital radiographs (Opportunity–Environmental Context and Resources): Obviously, the availability of digital radiographs is required to seamlessly use an AI-based diagnostic radiographic assistance tool. Six of the eight interviewed dentists had such digital radiographs available. Digitalization via scanning of analog films was found too time consuming.

Time pressure (Opportunity–Environmental Context and Resources) and legal requirements (Motivation–Reinforcement): Three dentists highlighted the legal need for a full systematic report, which is time consuming to generate and hence often abbreviated or written-up after the clinical work at the end of the day. AI applications may relieve dentists from this by automating the report generation and were expected to be time saving.

D1: “On the other hand, it also makes work easier, right./So, I mean, diagnosing of an OPG (panoramic radiograph)/, if you do it properly, you can sit for a quarter of an hour/, if you then put it down in writing/.”

D4: “If you could do that with one “click”, that would be good, right./because I know that from colleagues who studied together with me and who work as employed dentists/, there it is the rule/./and who then always groan that they have to sit down to write down these X-ray findings and diagnosis even after work.”

D4: “Yes, because I have to say/, it is actually a requirement to write down the detailed X-ray diagnosis. We just do not do that/, not out of laziness/, we just write the file briefly: why we did something and what can be seen, right. But with an OPG (panoramic radiograph) in particular, a lot more has to be diagnosed and put down in writing, right. And maybe that (the AI-tool) would be a support, because I know that you actually have to do that. And I actually think it is stupid that we do not do that.”

Improved accuracy (Motivation–Beliefs about Consequences): Three dentists reported on the difficulties of radiographic diagnostics and the associated uncertainty, especially towards caries diagnostics on bitewings. Improving and standardizing this accuracy was seen as a benefit.

D1: “/so I am not claiming that I always see everything. I am only human too. As a human being, you overlook things, and such an incorruptible algorithm is sometimes very useful. There are situations/, a patient comes along and has problems/, and then you can interpret a little more in such a picture than there is. So also, not wrong for something like that.”

D4: “Well that always happens/, I already had. I do bitewings/, you do not see anything, but in the mouth, I have a suspicion/, then I drilled into it and there was caries.”

D5 (oral surgeon): “For us that would be, so to speak, always in relation to the question: ‘Are there any additional findings?’/, because that is not the focus we are looking at.”

D1: (Concerning AI) “/which of course means that there is a constant standard of diagnosis. And I think that makes perfect sense, right.”

Responsibility (Motivation–Social/Professional Role and Identity): One dentist highlighted that it is in his responsibility to provide a sound and detailed diagnosis and found that AI could help him reach this goal.

D5 (oral surgeon): “/we also have dentists who send us patients just to take an X-ray because they do not have the appropriate equipment/, and of course then it would be smart, when the software could analyze the picture and you just have to look over it again/. That would certainly make work a bit easier.”

### 3.2. Conflicting Themes for Dentists

Impact of AI (Opportunity–Environmental Context and Resources): Two dentists were not sure about the impact of AI on their own diagnostic approaches and feared to be biased by it.

D6: “The question is, will I be influenced in my decisions by the given suggestion. Do I want to control myself or does the program controls me?”

Reliance on AI and responsibility (Motivation–Social/Professional Role and Identity): Along the same line, four dentists expressed apprehensions that staff or dentists may (possibly long-term) rely too much on the AI. They were unsure if this was a good thing, supporting and relieving them, or not.

D3: “/that you can rely on it completely/, and only focusses on these marked areas/, and then maybe lose track of it yourself/somehow rest a bit on it (the program), saying: ‘Well, the program did not show me that and therefore I could not see it.’”

D4: (concerning reliance on AI-diagnose) “Yes you should not do that. So that is the responsibility of every individual dentist”.

D5: (concerning reliance on AI-diagnose) “/so the legal situation is clear/, and also the need to look at it (X-ray) again for yourself/, is also clear.”

One dentist highlighted the importance of remaining responsible and critical towards AI and requiring time to doublecheck AI findings.

D8: “Having time/, look up and thinking. Can there be something or is that a software error after all, or something that the software does not know.”

### 3.3. Barriers for Dentists

Fears of malfunction (Motivation–Emotion): Two dentists commented that they were afraid of malfunction of the AI. One dentist indicated that it would be relevant to spot such errors.

D4: “I would rather be afraid that the program might interpret something that is not there or something like that.”

D8: “/the question is whether the program is able to diagnose overlays as overlays and not as caries/. This could be a problem.”

D1: “I mean the question is: ‚What when you get false-positive results?’”

D8: “/having time/, look up and think. Can it be or is it a software error after all/, or something that the software does not know.”

Technological hurdles and fear (Opportunity–Environmental Context and Resources and Motivation–Beliefs about Consequences): One dentist thought the technology may not be easy to maintain and compared the AI tool to his usual radiographic equipment.

D2: “Documentation and maintenance and cost structure associated with it/. Beginning with the daily analysis of the monitor/, which probably nobody does/, but what is prescribed/, and so on, right. Until all the check-ups which are necessary are done/.”

Moreover, another noted that patients may fear AI as part of the dental diagnosis.

D7: “/when an apparatus, a machine, a computer program says: ‘That and that is going on, and that and that should be done, or that is the therapy recommendation.’ Then it is like in a science-fiction movie. And I mean, people are not ready for that yet.”

I: “So, you mean the dentist can use it for its own support, but not in front of the patient.”

D7: “Exactly.”

### 3.4. Enablers for Patients

Digital literacy (Capability–Knowledge): Patients with an engineering background, who understood the technology, showed high confidence into AI and its usefulness.

P4: “I see everything that offers automated support in a relatively positive way. So, I also work with programs that somehow support you/, why should not you use that. I think support is always good.”

Understanding the reasoning behind the AI (e.g., having an element of explainability) was also found an enabler.

P2: “But if you are prepared for the fact that there can be mistakes/, and then asks again why he shows that/, and then maybe you can see for yourself why he is displaying it/, that understands. Maybe a program could write that too: ‘Because of this thing I marked it that way’. That would be ideal.”

Social experience with AI (Opportunity–Social Influences): Four of five patients found any experience of their dentists or their family with AI to be enablers, strengthening their trust.

P5: “If there is any suspicion and the dentist would like to check the X-ray also with the help of the AI, then I would follow the dentist recommendation, of course.”

All interviewed patients were positive towards practices providing AI, seeing the potential for improving care. They also found that practices taking up this technology may be seen as more innovative and up to current standards.

I: “Would you prefer a dental practice using the AI-tool?”

P1: “Potentially yes, because it shows that the doctor is ready to innovate and up to date.”

Costs and communication (Motivation–Reinforcement): Four of five patients stated that they would pay for an AI tool be used for their radiographic diagnostic independently from any health insurance. Four of five patients would also like to receive an AI-augmented, comprehensive medical report, as a “second opinion” (P1, 4/5).

P1: “I had to request it (the X-ray pictures). Although my former practice also caused problems. They only printed the X-ray picture, what is not sufficient for a second opinion at another dentist. That was a pretty heck of a mess.”

P4: “Yes of course. So that you can understand that better as a patient. That would be very helpful.”

P5: “Exactly, with such a report you could go to another dentist and get a second opinion. This would be fantastic, right.”

Improved accuracy (Motivation–Beliefs about Consequences): Four of five patients described negative experiences with dentists before, facilitating their hope that AI-based diagnostics would benefit them in the future by increasing diagnostic accuracy.

I: “And then the dentist could not really identify where the problem is (on the X-ray picture)?”

P2: “No. What you might have seen on the X-ray. But the pictures are sometimes not so easy to interpret.”

I: “Has it already happened that you had complaints, and nothing could be determined?”

P3: “Yes, that happens a lot/, there are also many mistakes. When they say: ‘You need two or three more root canal treatments’ or ‘here you have a problem in addition. You need a filling too.’/fifty percent (of the dentists) lie. That is just the way it is.”

### 3.5. Conflicting Themes for Patients

Responsibility for an adequate diagnosis: Three patients understood the technology behind AI and that AI can fail (Capability–Knowledge). They hence demanded dentists to not fully rely on the AI only.

P1: “/Nowadays it doesn’t quite get across that the computer can also make mistakes. And that should be clear.”

P2: “/it has to be clear for all persons that deep learning programs also like to make mistakes that a person would not make.”

P4: “/you cannot rely on it one hundred percent because they are just machines. We (place of work from P4) still have an authority over it, a person who then counterchecks it.”

Three of five patients indicated that it would be unsettling if staff or dentists rely too much on the software. One patient mentioned it would also be unsettling to not know if dentists spot possible malfunctions and are able to correct them (Motivation–Social/Professional Role and Identity).

P2: “/it must be clearly stated (to dentists who use the AI-tool) that you always have to think again about what you are doing/, that you should not rely blindly on the program./and then it should be clear: ‘Here the computer is usually a little bit better than our dentists. The program marks it.’ But it should just be clear to everyone that there might be mistakes/, and do not take it as face value directly.”

P1: “Of course, it can be that patients are unsettled (by the use of AI)./because things are potentially marked that may not need treatment after all or mistakes occur by the program. When mistakes happen, patients could say: ‘But why aren’t you doing anything now, everything is red. ‘To argue against the program is difficult then, because for the patient, the dentist gives a subjective and the software an objective opinion.”

### 3.6. Barriers for Patients

Validation and training (Capability–Knowledge): One patient mentioned that extensive regulatory validation of any AI and training of dentists and staff would be needed prior to it being used.

P1: “When a practice test has taken place and the dentist is familiar with it, so to speak.”

The presentation and juxtaposition of themes in Figure 2 revealed that there are some matching themes between dentists and patients. Experiences with AI by colleagues or family members, the option to generate a detailed digital medical statement using AI, the chance of an improved accuracy and, generally, an openness for new technologies were main enablers for both stakeholders. Main conflicting themes were that dentist may fully rely on an AI tool, thus suffering from bias and lacking accountability, as well as worries around data protection. A main barrier was the fear that malfunctions of the AI software may not be detectable for dentists and go unnoticed.

## 4. Discussion

Applications of AI are entering dental practice; providers and receivers of dental care will soon be confronted with AI. The aim of our study was to assess the attitude of dentists and patients towards AI for dental diagnostics, with a focus on radiography, as well as to identify barriers and enablers for implementing AI in clinical dentistry. A multi-perspective qualitative study was conducted and established theoretical instruments were employed for comprehensive and systematic analysis. In total, 36 enablers, barriers and conflicting themes were identified, covering nine of the domains of the TDF, and touching all three relevant behavior determinants, capability, opportunity and motivation. Overall, we found both stakeholder groups, dentists and patients, to be positive and open towards AI for dental diagnostics, noting the chances for a higher diagnostics quality, a relieved daily workload and a better communication and decision-making. Notably, dentists did not show any worries that AI applications could one day take over a significant amount of work and responsibility. This may well be as dentists’ tasks are heterogeneous, with only a minority allowing automation using AI, at least with the current state-of-the-art technologies. Some medical specialist such as radiologists, who do not directly communicate with patients (requiring empathy which machines do not have), do not contrast their findings against clinical signs and symptoms (which is standard in dental care to come to diagnosis and therapy decision) and do not perform the subsequent therapy, are expected to undergo significant changes in their daily work induced by AI [42]. Fewer changes by AI implementation are likely for other physicians, dentists among them [1,43,44,45,46]. Notably, dental radiologists (a specialty which does not exist in Germany) may have brought up other arguments than the practitioners interviewed, something to highlight at this point.

Several aspects were identified as enablers for implementing AI. First, and from a dentist’s perspective, the option to generate an automated diagnostic report after interaction with AI findings, thereby saving time for reporting, was a relevant enabler. This was associated with interviewees acknowledging this task as legally required but labor-intensive and automatable. Patients found the generation and receipt of such report desirable to obtain a full documentation of their oral health status, also for communication with other healthcare professionals. Second, both stakeholders expected AI to increase the diagnostic accuracy. Such expectations were more pronounced in patients, especially those with experience in invasive dental work. Important enabler for patients is that AI provides a second independent opinion and gives more confidence into the diagnosis of the dentist. Third, this trust into the AI was enhanced by the option to improve communication. Prior to the interviews, we demonstrated how current software frontends look and work. Patients and dentists further evaluated exemplary images of radiographs which had been assessed by AI models and which highlighted pathological findings on the radiograph in colors. For patients, this greatly increased their understanding of the radiograph and their trust into the subsequent diagnostic and therapeutic steps.

All these enablers were closely related to a general technological openness. Individuals with technological expertise or background showed a more positive attitude and expected AI applications to mature with time. The identified enablers align well with those found for AI applications in radiology, for instance the work in [43], where the authors interviewed radiologists, healthcare managers and industry stakeholders. Some important enablers were, as in the present study: (i) improved diagnostic quality and avoidance of mistakes; (ii) reduced workload by time-saving; (iii) more consistent reporting; (iv) having the AI integrated in existing routines and clinical pathways; and (v) openness towards AI applications [43,44].

As conflicting themes, we identified those around issues of reliance, responsibility and safety. Obviously, all parties were aware that AI applications will not be free of error potential and were interested to understand how practitioners would deal with this. If an erroneous AI finding could be spotted and corrected, this greatly increased the trust of patients. Our findings highlight the importance of explainable AI in the medical domain. In line with this, patients expected their dentists to maintain control and carry the final responsibility for any diagnostic finding, being able to de-bug or override the AI. This is notable, as the same was demanded by dentists, who found this responsibility sitting with them as part of how they perceive their professional role. Both sides also agreed that technological competency but also training with each specific AI tool was required to cover this requirement. If such training was not granted or an AI tool used which has not been sufficiently validated (which is unlikely as most AI tools for diagnostic purposes are regulated medical products), these conflicting themes were acting as barriers. The relevance of understanding the basic principles, potentials and limits of AI to ensure quality and safety assessment has been found in other study, as has the importance of ensuring or scrutinizing AI results and reliability [43,47,48,49,50].

A similar intermediate position was found for data protection aspects. If these were addressed sufficiently and in an accepted manner, data protection was not necessarily a barrier. If, however, dentists or patients perceived an AI application as not fully safeguarded against data protection breaches or legal conflicts, this was a clear barrier.

Another barrier was related to the previous point. Both patients and dentists feared errors of the AI not being identifiable and leading to wrong therapeutic decisions. The importance of explainability has been confirmed previously [43]. Certain stakeholders grounded this in having heard of AI making systematic errors, mainly stemming from bias in the underlying data pool [47,51,52], something which has been discussed in mainstream media, too [53]. Addressing these fears appropriately will be a major task in AI research and development, especially in front of discussed limitations of generalizability of dental AI applications [54].

Generally, this study comes with a range of strengths and limitations. First, we assessed the attitudes and expectations of two major stakeholder groups, dentists and patients, towards dental AI diagnostics, a timely field with growing research and clinical relevance. Contrasting insights from different stakeholder is useful and yields a more comprehensive picture of possible barriers and enablers and also facilitates triangulation. Second, we employed the TDF and associated instruments, strengthening the foundation of this study and increasing our analytic comprehensiveness and rigor. The option to deduce interventions may be used by future studies or stakeholders such as policymakers or industry in the AI field. Third, and as a limitation, our sample was small and, as usual for qualitative studies, not representative. Despite sampling care providers and patients from different backgrounds and of different age or gender, this sample cannot claim representativeness. Our findings will not necessarily be generalizable. In addition, our interviews focused on AI for 2D radiography; we did not explicitly ask for 3D radiographs and hence cannot infer in detail on this topic. This was done as very few German dentists own or use a 3D radiographic device or have the license to operate one. Notably, dentists have expressed a high need for support especially for this type of imagery, and our findings may have differed if particularly inquiring users of 3D radiographic devices [55,56]. Last, our findings are situated in the German healthcare context and specifically the field of dental diagnostics; transferring them to other settings or use cases should be done with caution. Notably, we expect those barriers and enablers unrelated to the specific German healthcare organization to be somewhat transferable, as attitudes and expectations towards medical diagnostics may be similar across use cases (at least in dentistry) or settings (at least in technologically advanced settings, e.g., dental practices in most high-income countries).

## 5. Conclusions

Both stakeholders emphasized the chances and hopes for AI. A range of enablers for implementing AI in dental diagnostics were identified, e.g., the chance for higher diagnostic accuracy, a reduced workload, more comprehensive reporting and better patient–provider communication. Barriers related to reliance on AI and responsibility for medical decisions, as well as the explainability of AI and the related option to de-bug AI applications, emerged. Decision-makers and industry may want to consider these aspects to foster implementation of AI in dentistry.

## Data Availability

The data used in this study cannot be made available by the authors given data protection rules.
